# Sinomenine pretreatment alleviates hepatic ischemia/reperfusion injury through activating Nrf‐2/HO‐1 pathway

**DOI:** 10.1002/iid3.700

**Published:** 2022-09-28

**Authors:** Bo Hui, Yantao Shu, Dandan Yang, Zhidong Wang, Li Zhang, Nina Lei, Zhengan Yang

**Affiliations:** ^1^ Department of General Surgery Unit‐4 The Second Affiliated Hospital of Xi'an Jiaotong University Xi'an Shaanxi China

**Keywords:** hepatic injury, inflammation, ischemia reperfusion, Nrf‐2, oxidative stress

## Abstract

**Introduction:**

Ischemia–reperfusion (IR) injury is induced by an interrupted blood flow and succeeding blood restoration, which is common in the operation of liver transplantation. Serious IR injury is a major reason leading to transplant failure. Hepatic IR is featured by excessive inflammatory response, oxidative stress, and apoptosis. Sinomenine (SIN) is derived from the herb Sinomeniumacutum and shows properties of anti‐inflammation and antiapoptosis in multiple IR‐induced organ injuries. However, the effect of SIN in hepatic IR has not been investigated.

**Methods:**

This study aims to investigate impacts of SIN on hepatic IR and the involved signaling pathway. An in vivo rat model of syngeneic orthotopic liver transplantation was constructed to induce the hepatic IR injury.

**Results:**

Results showed that SIN pretreatment provided a significant prevention against IR‐induced hepatic injury as manifested by the downregulated activities of serum alanine aminotransferase, aspartate aminotransferase, and lactate dehydrogenase, the alleviatedoxidative stress as shown by increased activities of serum superoxide dismutase and glutathione peroxidase, and decreased serum level of malondialdehyde, the suppressed inflammatory responses as shown by downregulated serum tumor necrosis factor‐α, interleukin (IL)‐6, IL‐8 levels, and upregulated IL‐10 level, as well as attenuated apoptosis as shown by decreased protein expression of cleaved caspase‐3 and −9. In line with these results, SIN pretreatment also alleviatedthe hepatic histopathological changes in IR rats and induced Nrf‐2/HO‐1 activation. The use of brusatol, a selective inhibitor for Nrf‐2, effectively reversed SIN‐induced above effects.

**Conclusions:**

Altogether, our results demonstrate that SIN might be a useful therapeutic drug for preventing hepatic IR‐induced injury during clinical liver transplantation.

## Graphical abstract

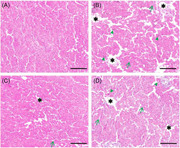

## 1 INTRODUCTION

Liver transplantation is currently the best therapeutic treatment for various advanced liver diseases due to the rapid progressin surgical skills and immunosuppressive drugs.^1^ Patients suffering from liver transplantation have an average 1‐year survival rate above 80%.^2^ However, challenges still exist and need urgent resolution. Ischemia‐reperfusion (IR), as the most influencing factor during liver transplantation, induces ischemic liver injury and succeeding reperfusion damage.^3^ The liver ischemic damage is featured by the consumption of adenosine triphosphate (ATP) and glycogens, as well as metabolic stresses resulting from the dysfunction of mitochondria, which finally induces cell death.^4^ While succeeding reperfusion damage is characterized by sustained severe hepatic damage caused by blood flow and reoxygenation.^4^ Hepatic IR‐induced tissue damage includes a series of pathological courses, during which metabolic disorders, excessive pro‐inflammatory cytokines, as well as reactive oxygen species (ROS) trigger a severe inflammatory cascade reaction, which is highly possible to cause the failure of transplantation.^5^

Sinomenine (SIN) is one kind of alkaloids and isolated from the traditional medical herb Sinomeniumacutum. It shows multiple pharmacological benefits, involving anti‐oxidation, anti‐inflammation, immunosuppression, analgesia, as well as antiapoptosis.^6^ A recent study found that SIN protected neurons against oxidative stress and cytotoxicity through ROS‐dependent increase of endogenous antioxidation.^7^ Another study showed that SIN effectively alleviated sepsis‐induced acute lung damage by inhibiting inflammation and oxidative stress.^8^ Additionally, SIN exerted the anti‐inflammation and renal protective roles via activation of Nrf‐2 signaling.^9^ According to the above findings, we assumed that SIN might be effective against IR‐induced hepatic injury and could be potent to protect hepatocytes against apoptosis after IR insult. Here, the hepatic protective roles of SIN against IR damage and its related mechanism of action were investigated.

## 2 MATERIALS AND METHODS

### 2.1 Experimental animals

Adult male Sprague‐Dawley rats (license number: SCXK (Shan) 2018‐001) at 8 weeks old weighing between 200 g and 250 g were purchased from the Experimental Animal Center of Xi'an Jiaotong University (XJU). Rats were kept under standard conditions at the temperature of 25°C, relative humidity of approximately 65%, with a cycle of 12h light/12 h dark. Rats got the standard chow and water freely. The use of rats and related experimental procedures were acknowledged by the Animal Care and Use Committee of the Second Affiliated Hospital of XJU, and strictly followed the Guide for the Care and Use of Laboratory Animals published by the National Research Council (USA).

### 2.2 Treatment of rats with SIN

Forty rats were grouped at random as follows: (I) the sham operation (*n* = 10); (II) Hepatic IR‐only (*n* = 10); (III) IR with SIN pretreatment group (*n* = 10); (IV) IR with SIN and BRU group (*n* = 10). SIN (98% purity, Sigma‐Aldrich) was freshly dissolved in sterile saline. The prepared SIN at 100 mg/kg was administered into rats via i.p. injection once per day for 5 consecutive days before IR induction. The dose of SIN was selected based on several previously published works.^10^, ^11^ Animals in Groups I and II were injected with normal sterile saline at the same frequency. Brusatol (BRU), a selective inhibitor of Nrf‐2, was commercially obtained and utilized to verify the involvement of Nrf‐2 signaling. The dose of BRU at 0.4 mg/kg was given intraperitoneally 1 hour before daily SIN administration for 5 consecutive days according to an early study.^12^

### 2.3 Hepatic IR induction

Animals received the environmental adaptation for 1‐week before experiments. Rats got anesthetized by using thiopental at a dose of 0.05 g/kg b.w. via intraperitoneal injection, followed by midline laparotomy. Hepatic IR induction was conducted according to an early study with minor modification.^13^ Briefly, hepatic ischemia was induced by placing a microvascular clip and sustained for 1 h which was validated by the pale look of clamped lobes. Then the clip was carefully removed to allow 3‐h blood reperfusion. Rat blood was collected under anesthesia. Serums were isolated using the centrifugal method. Liver tissues were harvested after rats were euthanized by carbon dioxide asphyxiation. All surgical procedures were performed under sterile conditions.

### 2.4 Biochemical assays

The activity of aspartate aminotransferase (AST), alanine aminotransferase (ALT), and lactate dehydrogenase (LDH) in the serum was determined by using colorimetric assay kits purchased from Jiancheng Bioengineering Institute (Nanjing, China).

The serum activities of superoxide dismutase (SOD) and glutathione peroxidase (GSH‐Px), as well as serum concentration of malondialdehyde (MAD) were measured to evaluate the antioxidant effect after SIN pretreatment. SOD and GSH‐Px activities, and MDA levels in the serum were assessed by a spectrophotometer and corresponding kits bought from Jiancheng Bioengineering Institute (Nanjing, China).

### 2.5 Assessment of cytokine levels by enzyme‐linked immunosorbent assay (ELISA)

Tumor necrosis factor‐α (TNF‐α), interleukin (IL)‐6, IL‐8, and IL‐10 levels in the serum were determined by ELISA. The detailed experimental operations were conducted referring to the manufacturer's instruction manual. These ELISA kits were commercially obtained from Xitang Biotechnology Ltd. (Shanghai, China).

### 2.6 Western blot analysis

Total proteins in liver tissues were extracted using radioimmunoprecipitation assay (RIPA) lysis buffer. The concentrations of proteins were calculated by using BCA Protein Assay Kits (Beyotime Biotechnology). Then 30 μg of proteins for each sample were loaded onto 10% sodium dodecyl sulfate‐polyacrylamide gel electrophoresis gels and then transferred onto polyvinylidene fluoride (PVDF) membranes. Membranes were blocked with5% nonfat milk at 25°C, and then cultured with a specific primary antibody (see Table 1) at 4°C overnight. On the next day, the culture medium containing primary antibody was discarded, and the PVDF membranes were rinsed three times using Tris‐buffered saline containing 0.05% Tween 20 (TBS‐T) and further cultured with horseradish peroxidase (HRP)‐conjugated secondary antibodies (see Table 2) for 1 h at 25°C. Finally, the protein bands were detected by using the enhanced chemiluminescence kits (Millipore) following the standard method. GAPDH served as an internal loading control.

**Table 1:** Primary antibodies used for western blot

Target gene	Catalog no.	Host	Vendor	Dilution
Cleaved caspase‐9	9507	Rabbit	Cell Signaling Technology	1:1000
Caspase‐9	9508	Mouse	Cell Signaling Technology	1:1000
Cleaved caspase‐3	9664	Rabbit	Cell Signaling Technology	1:1000
Caspase‐3	9662	Rabbit	Cell Signaling Technology	1:1000
Nrf‐2	33649	Rabbit	Cell Signaling Technology	1:1000
HO‐1	43966	Rabbit	Cell Signaling Technology	1:1000
GAPDH	sc‐365062	Mouse	Santa Cruz Biotechnology	1:200

**Table 2:** Secondary antibodies used for western blot

Secondary Ab.	Catalog no.	Vendor	Dilution
Bovine anti‐rabbit IgG‐HRP	sc‐2370	Santa Cruz Biotechnology	1:1000
Bovine anti‐mouse IgG‐HRP	sc‐2371	Santa Cruz Biotechnology	1:1000

Abbreviation:  HRP, horseradish peroxidase.

### 2.7 Statistics

All data analyses were conducted by using SPSS statistics version 20(SPSS Inc.). One‐way analysis of variance (ANOVA) was applied in multiple comparisons, while SNK‐*q* was further used in pair‐wise comparisons. Results were shown as the means ± standard deviation (SD). *p* < .05 indicates that the difference is statistically significant.

## 3 RESULTS

### 3.1 SIN reduces the serum levels of ALT, AST, and LDH in rats

**Figure 1:**
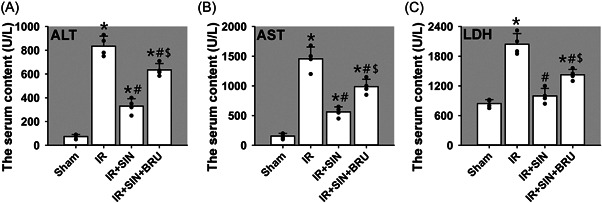
Hepatic injury was evaluated as release of transaminases (ALT and AST) and LDH in rat serum. (A) The serum ALT activity. (B) The serum AST activity. (C) The serum LDH activity. Bars represent means ± SD, *n* = 4 (the black dots represent the individual data points). ^*^ *p* < .05 versus sham operation group. ^#^ *p* < .05 versus IR‐only group. ^$^ *p* < .05 versus IR + SIN group. ALT, alanine aminotransferase; AST, aspartate aminotransferase; IR, Ischemia–reperfusion; LDH, lactate dehydrogenase; SD, standard deviation; SIN, sinomenine

We first evaluated liver functions by examining transaminases (ALT and AST) and LDH activity in rat serum. Results revealed that compared to those in the sham group, serum activities of ALT (Figure 1A) and AST (Figure 1B) in the IR‐only, IR + SIN, and IR + SIN + BRU (a potent Nrf‐2 inhibitor) groups were significantly upregulated (*p* < .05), serum activity of LDH (Figure 1C) in IR‐only and IR + SIN + BRU groups was upregulated (*p* < .05), while showing no change in IR + SIN group. Compared with those in IR‐only group, the serum ALT, AST, and LDH activities in IR + SIN and IR + SIN + BRU groups were reduced (*p* < .05). Compared with those in IR + SIN group, serum ALT, AST, and LDH activities in IR + SIN + BRU group were increased (*p* < .05). Above results preliminarily indicate a potential protective role of SIN against IR injury.

### 3.2 SIN relieves the oxidative stress in rat serum

**Figure 2:**
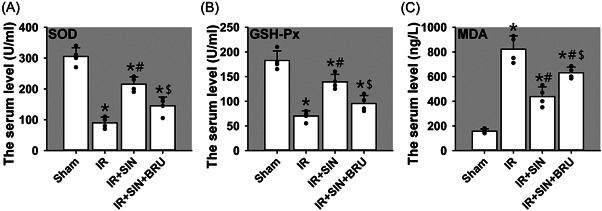
Roles of SIN in hepatic IR‐induced oxidative stress in rat serum. (A) The activity of SOD. (B) The activity of GSH‐Px. (C) The content of MDA. Bars show means ± SD, *n* = 4 (the black dots represent the individual data points). ^*^ *p* < .05 versus the sham operation group. ^#^ *p* < .05 versus the IR‐only group. ^$^ *p* < .05 versus IR + SIN group. GSH‐PX, glutathione peroxidase; IR, ischemia–reperfusion; MDA, malondialdehyde; SOD, superoxide dismutase; SD, standard deviation; SIN, sinomenine

Then we investigated the roles of SIN in IR‐induced oxidative stress. Results found that compared with the sham operation, SOD (Figure 2A) and GSH‐Px (Figure 2B) activities in IR‐only, IR + SIN, and IR + SIN + BRU groups were reduced (*p* < .05), while MDA level (Figure 2C) in IR, IR + SIN, and IR + SIN + BRU groups were elevated (*p* < .05). Compared to the IR‐only treatment, SOD and GSH‐Px activities in IR + SIN group were increased (*p* < .05), while showing no change in IR + SIN + BRU group, the content of MDA in both IR + SIN and IR + SIN + BRU groups was reduced (*p* < .05). Compared to IR + SIN treatment, SOD and GSH‐Px activities in IR + SIN + BRU group were reduced (*p* < .05), while MDA content in IR + SIN + BRU group was elevated (*p* < .05). Taken together, above data suggest an antioxidative effect of SIN on IR‐induced liver injury.

### 3.3 SIN attenuates the IR‐induced production of pro‐inflammatory cytokines while increases anti‐inflammatory cytokines in rat serum

**Figure 3:**
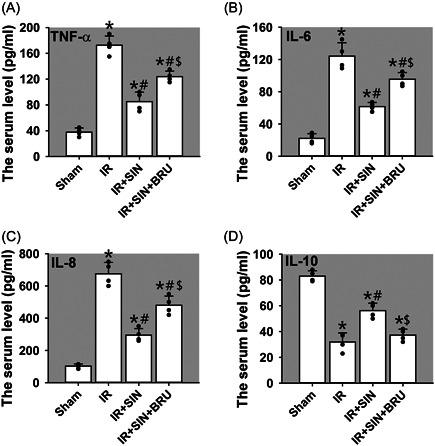
Roles of SIN in hepatic IR‐induced inflammatory response in rat serum. (A) The content of TNF‐α. (B) The content of IL‐6. (C) The content of IL‐8. (D) The content of IL‐10. Bars represent means ± SD, *n* = 4 (the black dots represent the individual data points). ^*^ *p* < .05 versus the sham operation group. ^#^ *p* < .05 versus the IR‐only group. ^$^ *p* < .05 versus the IR + SIN group. IL, interleukin; IR, ischemia–reperfusion; SD, standard deviation; SIN, sinomenine; TNF‐α, tumor necrosis factor‐α

The above results prompted us to examine the impact of SIN on the production of inflammatory mediators that are implicated in IR‐induced tissue damage.^14^ To this end, we selectively examined the serum levels of pro‐inflammatory mediators such as TNF‐α (Figure 3A), IL‐6 (Figure 3B), and IL‐8 (Figure 3C), also the anti‐inflammatory mediator IL‐10 (Figure 3D) by ELISA. It was noted that compared with the sham operation, serum TNF‐α, IL‐6, and IL‐8 levels in IR, IR + SIN, and IR + SIN + BRU groups were upregulated (*p* < .05), while IL‐10 level in IR, IR + SIN, and IR + SIN + BRU groups were decreased (*p* < .05). Compared with IR‐only treatment, TNF‐α, IL‐6, and IL‐8 levels in the IR + SIN and IR + SIN + BRU groups were reduced (*p* < .05), while IL‐10 level in IR + SIN group was increased (*p* < .05), showing no change in IR + SIN + BRU group. Compared with IR + SIN treatment, TNF‐α, IL‐6, and IL‐8 levels in IR + SIN + BRU group were elevated (*p* < .05), while IL‐10 levels in IR + SIN + BRU group was decreased (*p* < .05). Altogether, these data indicate that pre‐administration of SIN attenuates IR‐induced production of pro‐inflammatory cytokines, while promotes production of anti‐inflammatory cytokines.

### 3.4 SIN inhibits caspase‐9/caspase‐3‐dependent apoptosis in rat liver tissue

**Figure 4:**
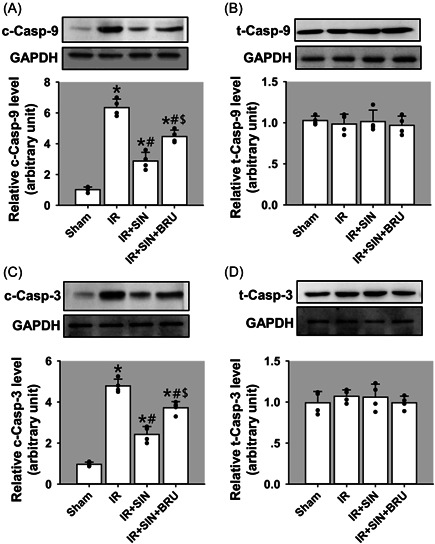
Roles of SIN in hepatic IR‐induced cell apoptosis in rat liver tissues. (A) The protein expression of cleaved caspase‐9. (B) The protein expression of total caspase‐9. (C) The protein expression of cleaved caspase‐3. (D) The protein expression of total caspase‐3. Bars represent means ± SD, *n* = 4 (the black dots represent the individual data points). ^*^ *p* < .05 versus the sham operation group. ^#^ *p* < .05 versus the IR‐only group. ^$^ *p* < .05 versus the IR + SIN group. IR, ischemia–reperfusion; SD, standard deviation; SIN, sinomenine

We further examined the expression of proapoptotic molecules, caspase‐9, and caspase‐3, by Immunoblotting. Results revealed that compared to the sham operation, the cleaved caspase‐9 (Figure 4A) and caspase‐3 (Figure 4C) in IR, IR + SIN, and IR + SIN + BRU groups were significantly induced (*p* < .05). Compared with the IR‐only treatment, the cleaved caspase‐9 and cleaved caspase‐3 in IR + SIN and IR + SIN + BRU groups were decreased (*p* < .05). Compared with IR + SIN treatment, cleaved caspase‐9 and cleaved caspase‐3 in IR + SIN + BRU group were upregulated (*p* < .05). It was noted that the total caspase‐9 (Figure 4B) and total caspase‐3 (Figure 4D) expression levels were not significant among four groups. Our data thus suggest that SIN protects the liver against IR‐induced apoptosis via caspase‐9/caspase‐3‐dependent pathway.

### 3.5 SIN pre‐administration attenuates the severity of hepatic IR damage in rats

**Figure 5:**
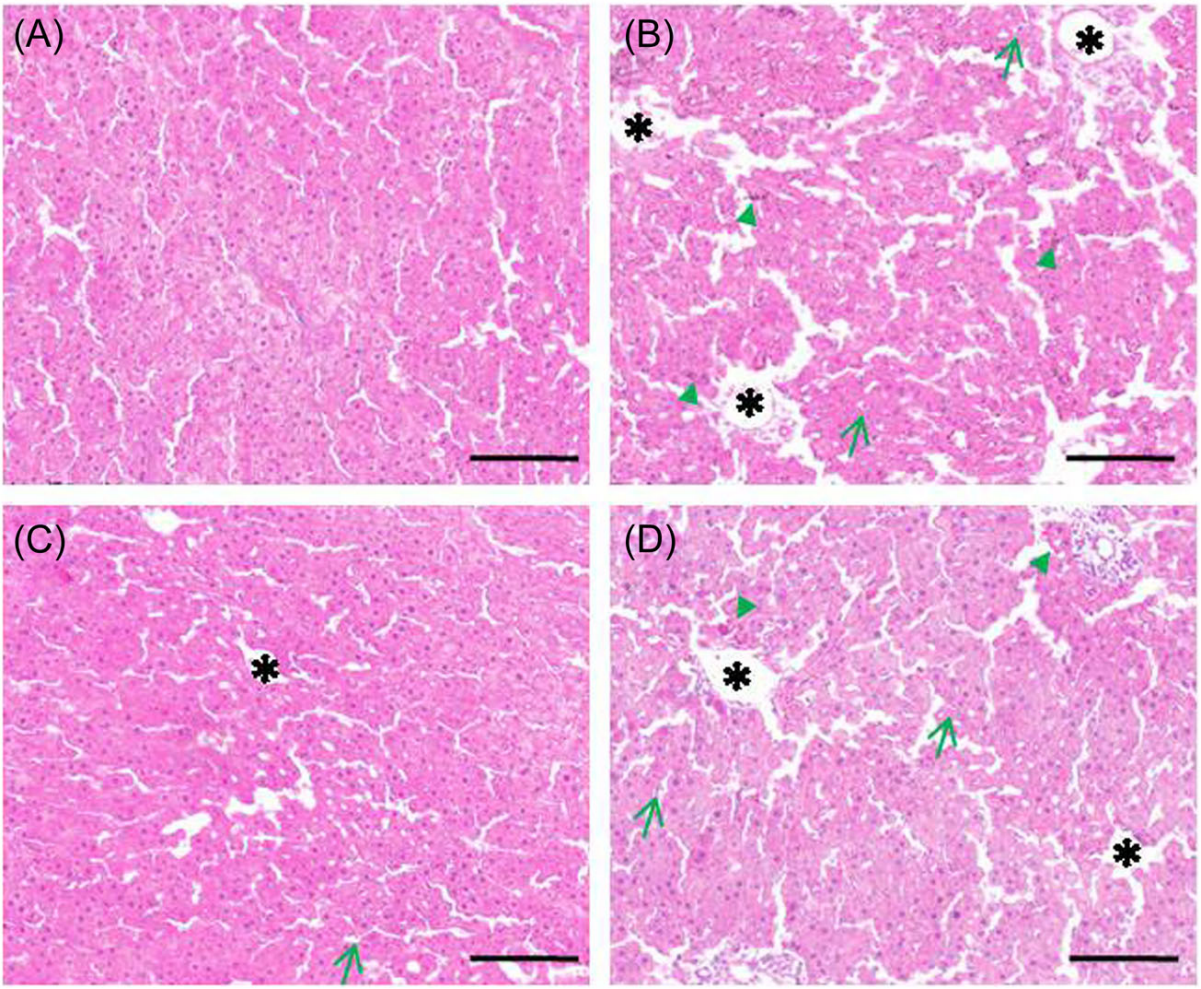
Roles of SIN in IR‐caused hepatic damage assessed by H&E staining. (A) The sham operation group. (B) IR‐only group. (C) IR + SIN group. (D) IR + SIN + BRU group. Green triangles indicate areas with inflammatory cell infiltration. Green arrows indicate areas with vacuolation. Black asterisks indicate necrotic areas. Scar bars = 150 μm. Images are under 100X magnification. BRU, brusatol; H&E, hematoxylin and eosin; IR, ischemia–reperfusion; SIN, sinomenine

H&E staining was conducted to analyze hepatic pathological alterations in rats receiving different treatments. Results showed that the hepatic sections in the sham group displayed the intact hepatic lobules without abnormal infiltration of inflammatory cells, disordered fragments, vacuolation, or massive necrosis (Figure 5A). However, the histopathological alterations in the three IR groups were different (Figure 5B‐D). IR significantly damaged hepatic morphology showing more inflammatory cell infiltration (Figure 5B, SEE *green arrowheads*), vacuole‐like changes (Figure 5B, SEE *green arrows*) and hepatocellular necrosis (Figure 5B, SEE *black asterisks*). In the SIN‐pretreated IR group, the liver lobule structure was almost complete, inflammatory cell infiltration was significantly alleviated, the proportion of cytoplasmic vacuoles and necrotic cells was greatly decreased (Figure 5C). With the use of BRU, the hepatic histopathological changes in SIN‐pretreated IR rats deteriorated again (Figure 5D).

### 3.6 SIN acts as an activator for Nrf‐2/HO‐1 signaling

**Figure 6:**
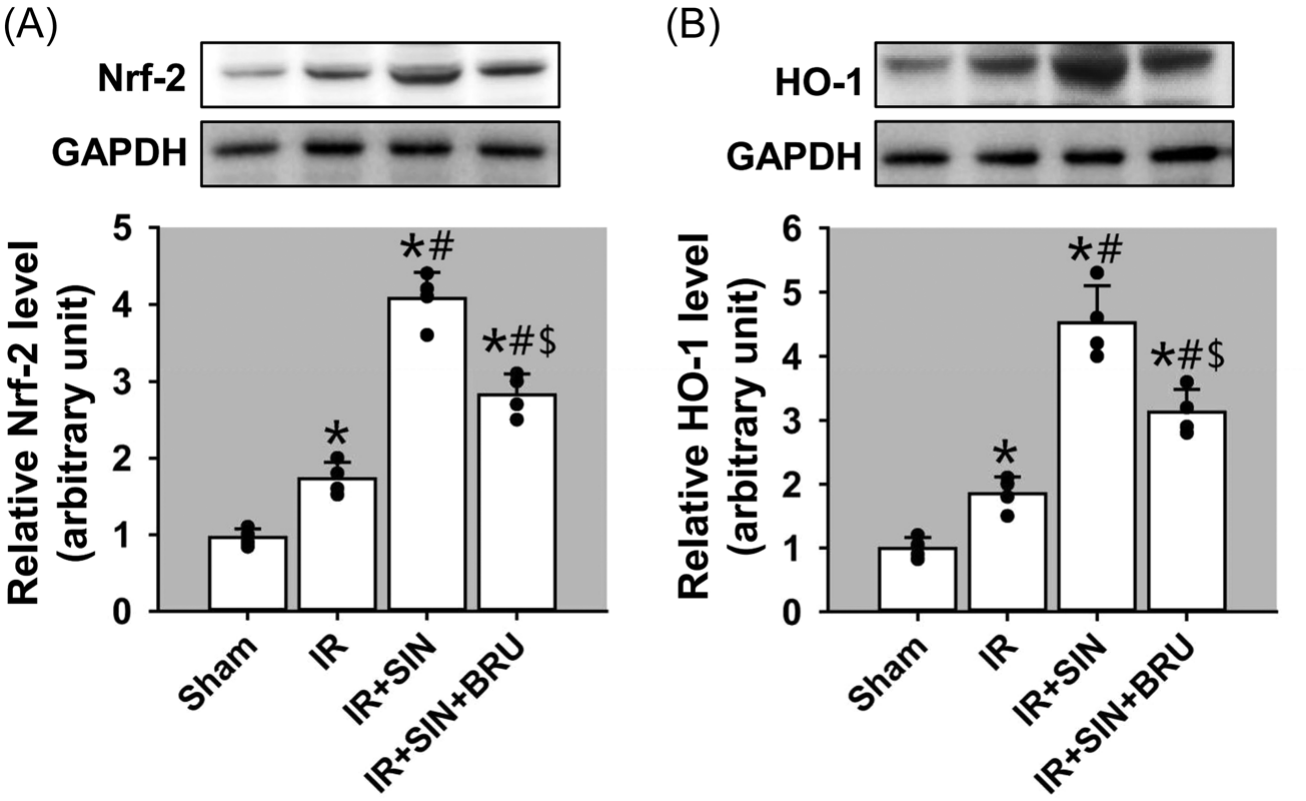
Activation of Nrf‐2/HO‐1 pathway in SIN‐mediated protection of hepatic IR injury. (A) The protein expression of Nrf‐2. (B) The protein expression of HO‐1. Bars represent means ± SD, *n* = 4 (the black dots represent the individual data points). ^*^ *p* < .05 versus the sham operation group. ^#^ *p* < .05 versus the IR‐only group. ^$^ *p* < .05 versus the IR + SIN group. IR, ischemia–reperfusion; SD, standard deviation; SIN, sinomenine

To clarify mechanisms through which SIN suppresses oxidative stress and inflammatory response, we examined its impact on Nrf‐2/HO‐1 signaling with the use of BRU, a potent Nrf‐2 inhibitor. To this end, we examined protein levels of Nrf‐2 (Figure 6A) and HO‐1 (Figure 6B) by Western blot analysis using liver tissues. Indeed, IR insult induced a moderate but significant increase of Nrf‐2 and HO‐1 protein levels (*p* < .05), suggesting activation of Nrf‐2/HO‐1 signaling. Remarkably, pre‐administration of SIN further augmented the upregulation of Nrf‐2 and HO‐1 (*p* < .05), indicating a further activation of Nrf‐2/HO‐1 signaling. However, BRU significantly suppressed Nrf‐2 and HO‐1 expression (*p* < .05), indicating attenuated Nrf‐2/HO‐1 signaling. Together, our data support that SIN is an activator for Nrf‐2/HO‐1 pathway.

## 4 DISCUSSION

Hepatic IR damage is a complex course induced by transient tissue deficiency of blood supply succeeded by blood reperfusion.^15^ It could happen under various clinical conditions including hepatic resection, trauma, shock, and liver transplantation.^15^ Liver transplantation is currently the optimal strategy to save the lives of patients suffering from advanced liver failure or malignancy. However, no effective therapeutics is available against peri‐transplant hepatic IR, which might lead to graft dysfunction, transplant rejection, and organ failure.^16^

In recent years, the research on herbal medicine has made great progress, about 80% of people worldwide use traditional herbal medicines as an important supplement for their general health care.^17^ The major active ingredients from herbal medicines have been applied to IR damage and obtained satisfactory results.^18^ However, these herbal medicines have complicated compositions and exert roles by targeting multiple pathways, including anti‐oxidation stress, anti‐inflammatory response, antiapoptosis, as well as suppressing the endoplasmic reticulum stress.^18^ The current study investigated SIN's protective roles in I/R‐induced hepatic damage and the involving mechanism.

First, we determined whether SIN pretreatment exerted a protective role in IR‐induced hepatic injury. Results showed that pre‐treating rats with SIN significantly attenuated hepatocellular damage as evidenced by down‐regulated hepatic injury markers (ALT, AST, and LDH), increased capabilities of anti‐oxidation and anti‐inflammation, weakened caspase‐medicated apoptosis, as well as further enhanced activation of Nrf‐2 signaling. These data indicated the significant protective role of SIN pretreatment in hepatic IR injury.

We next investigated whether SIN‐mediated protective role was related to the activation of Nrf‐2 signaling. Results found that the joint application of SIN and BRU, a putative suppressor of Nrf‐2, dramatically reversed the SIN‐induced protective effect as demonstrated by the elevated transaminase activities, increased oxidative stress, enhanced inflammatory response, aggravated apoptosis, as well as deactivated Nrf‐2/HO‐1 pathway. These data thus suggest SIN's protective role against hepatic IR injury was, at least partially, mediated through Nrf‐2 activation.

Oxidative stress is often induced by the imbalance between antioxidant defense system and excessive ROS production.^19^ IR could cause the excessive generation of various ROS, involving superoxide anion radicals, hydroxyl radicals, peroxynitrite, and hydrogen peroxide (H_2_O_2_).^20^ These excessive ROS further accelerate the peroxidation of lipids and generation of oxidant MDA, an important end product of lipid peroxidation.^21^ Moreover, IR could decrease the activity of many antioxidant enzymes like SOD and GSH‐Px. SOD is a mitochondrial matrix enzyme that scavenges superoxide anions,^21^ while GSH‐Px is the major antioxidant enzyme that protects cells from oxygen‐free radical‐induced injury and H_2_O_2_ assaults.^21^ Here we confirmed that SIN pretreatment remarkably reduced the serum MDA content via enhancing SOD and GSH‐Px activities. While the use of BRU obliviously attenuated SIN's protective effect against oxidative stress, indicating that SIN suppresses hepatic IR‐induced oxidative stress via Nrf‐2 signaling.

Inflammation can be the most severe secondary damage suffered in hepatic IR.^22^ Inflammatory cells that are abnormally activated can release abundant cytokines and cause further liver damage.^22^ TNF‐α is a critical initiator during inflammatory response, which can stimulate the transformation of monocytes into macrophages, and promote an inflammatory response.^23^ IL‐6 and IL‐8 are also critical pro‐inflammatory mediators during acute reacting period.^24^ Once the acute inflammation comes up, IL‐6 and IL‐8 increase promptly and initiate an inflammatory cascade reaction, therefore, IL‐6 and IL‐8 levels are important indicators that can reflect the inflammatory degree.^24^ IL‐10 is the best studied anti‐inflammatory cytokine.^25^ In this study, SIN pretreatment significantly reduced hepatic IR‐induced production of TNF‐α, IL‐6, and IL‐8, while increasing the serum IL‐10 level. BRU application abolished the effect of SIN on inflammation, demonstrating that SIN pre‐conditioning inhibits hepatic IR‐mediated inflammation response.

Another possible mechanism of liver IR damage is apoptosis. It is known that the execution of apoptosis is predominantly fulfilled via activating the caspase‐mediated pathway. Caspase‐8, −9, and −12 are known to be the key molecules in three apoptotic pathways, that is, extrinsic death receptor pathway, intrinsic pathway, and endoplasmic reticulum stress‐mediated pathway,^26^ respectively. All three caspases could subsequently promote caspase‐3 activation.^27^ In this study, cleaved caspase‐9, and cleaved caspase‐3 dramatically increased after IR insults, SIN pretreatment significantly suppressed these increases. While the use of BRU reversed the effect of SIN on the activation of caspase‐9 and caspase‐3, implying that SIN reduces hepatic IR‐induced apoptosis through Nrf‐2 signaling.

The cellular and molecular mechanisms underlying Nrf‐2‐mediated protection have been found to be related with the regulation of inflammatory response, the resistance of oxidative stress, and inhibition of cellular apoptosis. Nrf‐2 is an inducible transcription factor that is involved in various defense mechanisms. At the quiescent phase, Nrf‐2 normally exists in the cytosol.^28^ Upon the stimulation by oxidative substances, Nrf‐2 migrates into the nucleus, combines with a promoter, causes the release of downstream genes, and regulates the transcriptional activity of certain antioxidant enzymes and phase II metabolizing enzymes,^28^ which further eliminate the intracellular ROS and pro‐inflammatory cytokines, decrease serum MDA concentration, displaying the potent antioxidative and anti‐inflammation effects.^28^ As one of the major downstream genes of Nrf‐2, HO‐1 is inducible and also involved in oxidative stress.^28^ Nrf‐2/HO‐1 signal has been shown to be activated in sulforaphane‐induced prevention from hepatic IR damage.^29^ Additionally, Nrf‐2 activation has been identified as the molecular mechanism through which SIN exerts its anti‐inflammation, as well as the renal^9^ and cerebral^30^ protective functions. Moreover, Nrf‐2 signaling has participated in SIN‐induced protection against septic‐associated lung injury by regulating inflammation and oxidative stress.^8^ In this study, IR promoted Nrf‐2 and HO‐1 expression, implying that hepatic IR can promote the activation of Nrf‐2/HO‐1 signaling and enhance cellular antioxidant capacity. The use of SIN further enhanced Nrf‐2 and HO‐1 expression. However, BRU significantly reversed SIN's function in Nrf‐2/HO‐1 activation, suggesting that SIN promotes the expression of antioxidant proteins, and reduces hepatic IR‐induced oxidative stress and inflammatory response possibly through the activation of Nrf‐2 pathway.

This study elucidated that SIN exerted protective roles against liver IR damage in an in vivo rat model. The oxidative stresses, inflammatory responses, and cell apoptosis were alleviated in SIN pretreated rats subject to hepatic IR damage. Thus, SIN might serve as a potential therapeutic drug to treat liver IR damage. However, several matters need to be resolved before SIN's clinical application. For example, what is the underlying mechanism mediating the inductive effect of SIN on Nrf‐2 expression? Would this inductive effect on Nrf‐2 be also applied to humans? Would pre‐administration of SIN produce effective and efficient protection against IR injury during liver transplantation? Further research works are urgently needed to answer these questions.

## AUTHOR CONTRIBUTIONS

Bo Hui, Yantao Shu, and Dandan Yang performed animal experiments and analyzed data. Bo Hui, Zhidong Wang, and Li Zhang conducted literature research. Bo Hui and Nina Lei provided the reagents. Zhengan Yang and Bo Hui conceived the study and wrote the manuscript.

## CONFLICTS OF INTEREST

The authors declare no conflicts of interest.

## Data Availability

Data are available upon reasonable request.
